# Histone Deacetylase BpHST1 Regulates Plant Architecture and Photosynthesis in Birch

**DOI:** 10.3390/biology14121689

**Published:** 2025-11-27

**Authors:** Lili Hou, Baoxin Li, Mengyan Ge, Zhimin Zheng

**Affiliations:** 1 State Key Laboratory of Tree Genetics and Breeding, College of Forestry, Northeast Forestry University, Harbin 150040, China; 2 The Center for Basic Forestry Research, College of Forestry, Northeast Forestry University, Harbin 150040, China

**Keywords:** epigenetics, histone acetylation, *BpHST1*, plant architecture, birch

## Abstract

(1) Background: Epigenetic mechanisms play a significant role in plant architecture. Histone deacetylases, as crucial epigenetic regulators, shape plant architecture by modifying chromatin structure and regulating gene expression. (2) Methods: This study combined bioinformatic identification of *BpHST1* with its functional characterization in transgenic birch overexpressing *35S::BpHST1::FLAG*, including phenotypic and cytological analyses. The putative direct targets of *BpHST1* were further identified by integrating RNA-seq and ChIP-seq data. (3) Results: Phylogenetic analysis revealed that the *HST1* orthologs from birch and peach form a distinct clade, consistent with their high degree of protein sequence conservation. *BpHST1* exhibited light-inducible and leaf-preferential expression, with transcript levels elevated under light versus dark conditions, enriched in leaves relative to roots, and promoter activity confirming this spatial patterning. Overexpression of *BpHST1* significantly suppressed plant height, cell length, cell width, and photosynthetic capacity. Integrated RNA-seq and ChIP-seq analysis suggested that *BpLHCA2* possible functions as a direct downstream target of *BpHST1*, mediating plant growth and development. (4) Conclusions: Our findings delineated the role of *BpHST1* in regulating plant architecture through comprehensive expression and functional analyses, and identified a candidate target gene. This study provided a novel insight into the molecular mechanisms governing plant architecture and offers potential strategies for future epigenetic breeding.

## Simple Summary

Epigenetic mechanisms significantly regulate the architecture of forest trees, enhancing their adaptability and resilience to climate change. This study investigated the histone deacetylase gene *BpHST1* (*Homologs of Sirtuins Two 1*) as an epigenetic regulator in birch. *BpHST1* expression was induced by light and enriched in leaves. Overexpression of *BpHST1* produced shorter plants with reduced cell size and photosynthetic performance. Through integrated RNA sequencing (RNA-seq) and Chromatin Immunoprecipitation sequencing (ChIP-seq), we identified the photosynthesis gene *BpLHCA2* (*Light-Harvesting Complex A2*) as a direct downstream target. These findings elucidate an epigenetic growth-regulation module in birch, providing a basis for breeding improved plant architecture.

## 1. Introduction

Epigenetic modifications serve as indispensable regulators of plant growth, development, and environmental adaptation [1,2]. Functioning as a master switch, they dynamically modulate chromatin architecture to fine-tune gene expression within complex regulatory networks [3,4,5]. For example, histone deacetylation plays a critical role in developmental processes: recent studies demonstrated that acetylation of Smc3 modulates chromatin stability through its ability to stabilize the cohesin regulator Pds5, thereby ensuring precise control of chromatin organization [6]. Similarly, in *Arabidopsis*, *HDA6* and *HDA19* participate in abscisic acid (ABA), jasmonic acid (JA), and ethylene signaling pathways, enabling adaptive responses to environmental changes [7,8,9,10]. Furthermore, loss of *HDA18* function disrupts root epidermal patterning, underscoring the importance of histone deacetylases in cellular differentiation and organogenesis [8,11,12]. These epigenetic mechanisms collectively underpin plant phenotypic plasticity throughout development and in response to environmental cues, influencing processes such as seed development, flowering, fruit maturation, and biotic and abiotic stress responses [13,14,15,16,17]. However, research on the epigenetic regulation of plant architecture remains limited and has predominantly focused on model crops such as rice and *Arabidopsis* [18,19,20].

Forests are under increasing pressure from global environmental change [21]. Although reforestation and afforestation efforts in tropical, temperate, and boreal regions help mitigate climate change via carbon sequestration, growing evidence indicates that epigenetic variation enhances phenotypic plasticity and adaptive capacity in long-lived trees [22]. Given rapid climate change, forest epigenetics provides promising pathways for biotechnology and breeding, facilitating the selection of adaptive traits to bolster ecosystem resilience and sustainable management [23,24]. Therefore, epigenetic applications in woody plants warrant further extensive exploration.

Plant architecture plays a critical role in determining the efficiency of resource acquisition, such as light, water, and nutrients, by individual plants and populations, factors that directly influence yield formation [25]. Current research on plant architecture remains predominantly focused on major crops like rice [26], wheat [27], maize [28], and soybean [29], with the goal of boosting yield through architectural manipulation. In forest trees, architecture is governed by a complex regulatory network formed by the interplay among genetic, environmental, and hormonal factors. This network integrates hormonal pathways (e.g., GA, BR, auxin), transcription factors (including *WRKY* and *NAC*), and specific regulators (such as *TAC1*, *LAZY1*, and *WEEP*) to translate genetic programs and environmental signals into morphological outcomes [30].

White birch (*Betula platyphylla* Suk.) is an ecologically vital pioneer species that has been the subject of relatively limited molecular research, with existing studies focusing primarily on stress tolerance [31,32]. We focus on further exploring how epigenetics plays a role in plant architecture regulation. Previous studies have confirmed that *HST1* (*Homologs of Sirtuins Two 1*) is an NAD-dependent deacetylase gene that affects changes in plant architecture, but the specific molecular regulatory mechanism remains unclear [33,34,35]. Our results demonstrated that *BpHST1* regulates the expression of the light-harvesting gene *BpLHCA2* (*Light-Harvesting Complex A2*) by influencing photosynthetic light capture capacity, ultimately affecting growth and development in birch. This study established a foundational framework for exploring the epigenetic mechanisms underlying plant architecture in trees.

## 2. Materials and Methods

### 2.1. Plant Materials and Cultivation

Birch seedlings were grown for four months in a controlled environment (22 °C, 16-h light/8-h dark). Tender leaves from two-month-old plants were harvested, snap-frozen in liquid nitrogen, and stored at −80 °C. Three biologically independent replicates were analyzed for both RNA-seq and ChIP-seq, ensuring robust and reproducible data.

### 2.2. Identification and Analysis of BpHST1 Gene in Birch

We performed the phylogeny of *HST1* using the maximum likelihood method in MEGA and aligned the closely related peach and birch orthologs to evaluate their sequence conservation [36]. The exon-intron structure of the gene was analyzed with the Gene Structure Display Server [37]. For the cis-element analysis, a 2122 bp genomic sequence upstream of the transcription start site of *BpHST1* was defined as the promoter region. Putative cis-regulatory elements within this promoter were predicted using PlantCARE and visualized with TBtools -II (version 2.3.63) [38]. Protein structure prediction was conducted using the AlphaFold Server 3 [39].

### 2.3. Total RNA Extraction

Total RNA was isolated from the samples using the RNAeasy Kit (Cwbio, Taizhou, China). RNA integrity and purity were verified by spectrophotometry (A260/A280 ratio: 1.9–2.1). Subsequently, first-strand cDNA was synthesized from the qualified RNA templates using the PrimeScript RT Reagent Kit (Takara, Tokyo, Japan), followed by quantitative real-time PCR (qRT-PCR) analysis.

### 2.4. qRT-PCR

qRT-PCR assays were conducted using the UltraSYBR One Step mix (CWBIO, Beijing, China) with gene-specific primers (Supplementary Table S1). Amplification and detection were performed on a QuantStudio^TM^ 3 Real-Time PCR System (Applied Biosystems, Carlsbad, CA, USA). The Tubulin gene served as an internal reference for normalizing transcript levels, and the relative expression of each target gene was determined using the 2^−ΔΔCt^ method [40].

### 2.5. Plasmid Construction and Plant Transformation

We ligated the *BpHST1* CDS (Coding DNA Sequence) amplified from DL-1 to the pCAMBIA1305.1-FlAG vector. We ligated the DL-1-amplified *BpHST1* promoter sequence to the pCAMBIA1301-LUC vector. Primers are shown in Supplementary Table S1. Positive lines were obtained through *Agrobacterium*-mediated embryonic transformation of mature birch zygotic embryo [41].

### 2.6. Scanning Electron Microscope

Sample preparation was performed as follows: young stem segments (<1 cm long) were fixed in 2.5% glutaraldehyde. Following fixation, samples were dehydrated through an ascending tert-butanol series (30%, 50%, 70%, 90%, and 100%), with 15 min per step. After dehydration, samples were solidified at 4 °C for 30 min and vacuum-dried. Finally, the dried specimens were mounted on conductive stubs, sputter-coated with gold, and observed for cellular morphology.

### 2.7. Photosynthetic Gas Exchange Measurements

Photosynthetic gas exchange was assessed with a Li-6800 portable system (LI-COR, Bourne, MA, USA) fitted with a 6800-01A chamber. Measurements were taken under set conditions: 400 ± 10 µmol mol^−1^ CO_2_, 1200 µmol m^−2^·s^−1^ PPFD, and 40% relative humidity. The mid-lamina portion of leaves from healthy, uniform plants was sealed in the chamber, and data were recorded upon stabilization.

### 2.8. RNA-Seq

RNA-seq was conducted on young leaves of two-month-old DL-1 and *35S::BpHST1::FLAG* lines with three biological replicates each. From the nine total RNA samples, high-quality libraries were prepared and sequenced via Illumina NovaSeq 6000 (Illumina, San Diego, CA, USA). We mapped the clean reads to the birch reference genome via the Hisat2 plugin in TBtools and applied FPKM-based quantification to identify differentially expressed genes (FDR < 0.01, |log2FC| ≥ 1) [38].

### 2.9. ChIP-Seq

ChIP was conducted using young leaves from two-month-old DL-1 and *35S::BpHST1::FLAG* seedlings, following an established method with adjustments [42]. Immunoprecipitation employed 10 μg of anti-FLAG antibody per reaction. After capturing immune complexes, DNA was purified through washing, elution, and reverse cross-linking, and the resulting samples were used for library construction and sequencing at Novogene (Beijing, China).

## 3. Results

### 3.1. Identified and Expressed of BpHST1 in Birch

**Figure 1:**
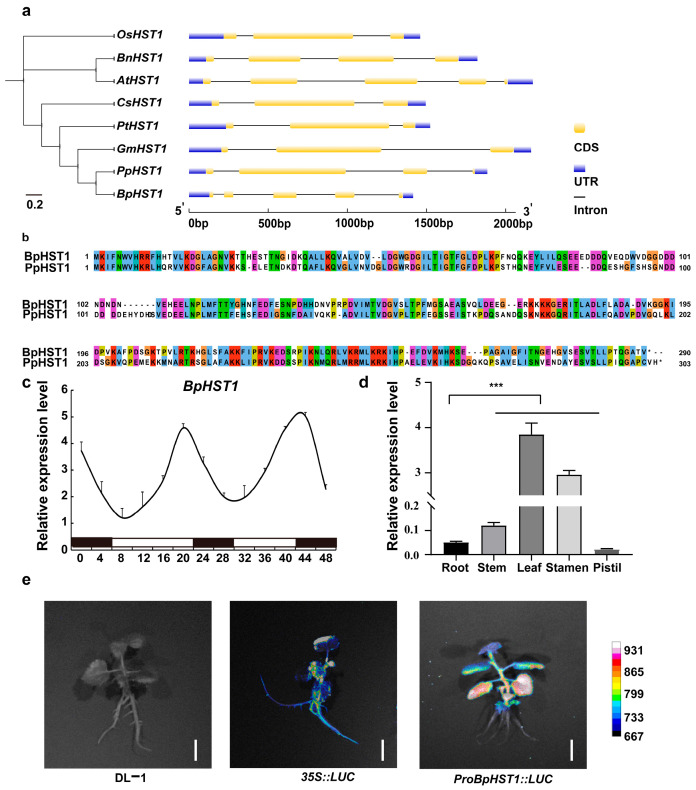
Bioinformatic characterization and expression analysis of the *BpHST1*. (**a**) Phylogenetic and gene structure analysis of *HST1* in different species. Depicting the arrangement of coding sequences (CDS, yellow boxes), untranslated regions (UTRs, blue boxes), and introns (lines). Os: *Oryza sativa*, Br: *Brassica rapa*, At: *Arabidopsis thaliana*, Cs: *Citrus sinensis*, Pt: *Populus trichocarpa*, Gm: *Glycine max*, Pp: *Prunus persica*, Bp: *Betula platyphylla*. (**b**) Amino acid alignment of the conserved domains of HST1 proteins from different organisms in birch and peach. * represents the stop codon of the protein. (**c**) Relative expression of *BpHST1* under light and dark conditions over a 48 h period. The black areas represent dark conditions. The white areas represent light conditions. Data are presented as the mean ± SD, *n* = 3. (**d**) Relative expression of *BpHST1* in various tissues of birch. Data are presented as the mean ± SD, *n* = 3. Asterisks represent significant differences (*** *p* < 0.001, *t*-test). (**e**) Analysis of *BpHST1* promoter activity in birch. DL-1 and *35S::LUC* as control. The pseudocolor bar showed the luminescence intensity in the image. Bar = 0.6 cm.

The histone deacetylase HST1 contains a conserved deacetylase-like domain and has been functionally characterized in species such as rice, *Arabidopsis*, and wheat. The consistent correlation between gene structures and phylogenetic clustering strongly supports the reliability of the current classification system for these genes. This integrated structural and evolutionary analysis provides important insights into the identification of *HST1* in birch (Figure 1a). The *HST1* orthologs from birch and peach clustered within the same phylogenetic clade, consistent with their high degree of protein sequence identity (Figure 1b). Promoter analysis of *BpHST1* revealed multiple cis-regulatory elements, indicating potential regulation by abscisic acid (ABRE), drought (MBS), and light (G-box) signals (Figure S1a). Furthermore, tertiary structure prediction of the BpHST1 protein showed a combination of α-helices and random coils, suggesting the potential for independently functional domains (Figure S1b). To assess the light responsiveness of *BpHST1*, we analyzed its expression in birch seedlings under dark and light conditions. The results showed that *BpHST1* was down-regulated in darkness and up-regulated in light (Figure 1c). Tissue-specific expression analysis further indicated pronounced accumulation of *BpHST1* transcripts in leaves, with minimal expression in roots (Figure 1d). The strong leaf-specific activity of the *BpHST1* promoter was confirmed by dual-luciferase reporter assays in birch (Figure 1e, Figure S1c).

### 3.2. Overexpression of BpHST1 Inhibited the Growth and Development in Birch

**Figure 2:**
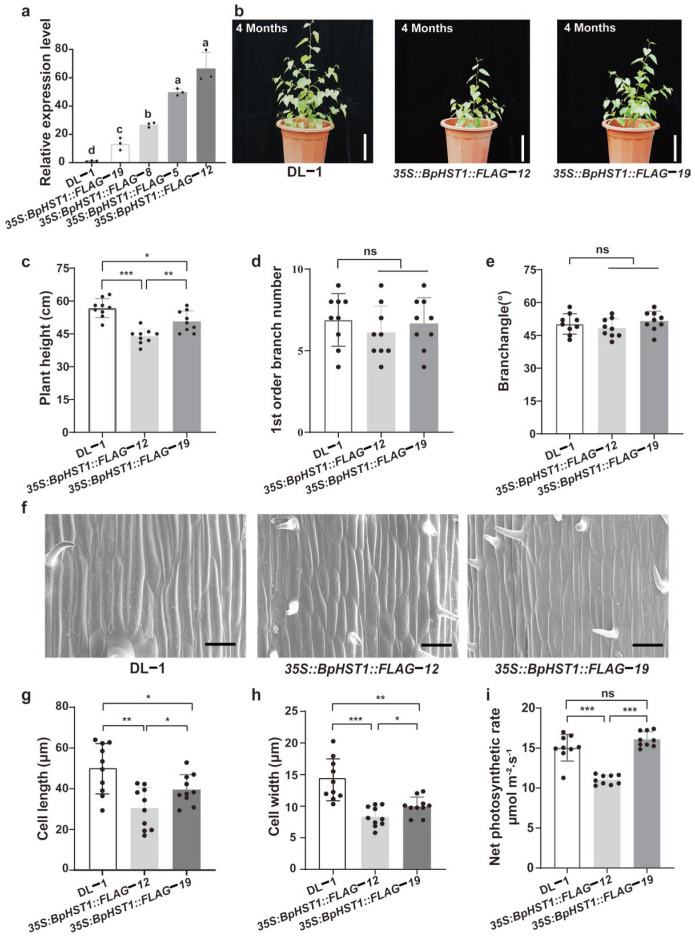
Phenotypic analysis of *BpHST1*-overexpressing lines. (**a**) Relative expression levels of *BpHST1* in transgenic lines. Data are presented as the mean ± SD, *n* = 3. Different letters indicate significant differences, by Duncan’s test at *p* < 0.05. (**b**) Phenotypic characterization of *35S::BpHST1::FLAG12* and *35S::BpHST1::FLAG19* lines. Bar = 10 cm. (**c**–**e**) Quantitative assessment of phenotypes: plant height, 1st order branch number and branch angle. Data are presented as the mean ± SD, *n* = 9. Asterisks represent significant differences (* *p* < 0.05, ** *p* < 0.01, *** *p* < 0.001, ns: not significant, *t*-test). (**f**) Scanning electron microscopy in *BpHST1* transgenic lines. Bar = 25 μm. (**g**,**h**) Quantification of cell length and width from scanning electron microscopy. Data are presented as the mean ± SD, *n* = 10. Asterisks represent significant differences (* *p* < 0.05, ** *p* < 0.01, *** *p* < 0.001, ns: not significant, *t*-test). (**i**) Quantification of the net photosynthetic rate in *BpHST1* transgenic lines. Data are presented as the mean ± SD, *n* = 9. (*** *p* < 0.001, ns: not significant, *t*-test).

To confirm the growth-regulatory function of *BpHST1*, we produced stable transgenic lines overexpressing a *35S::BpHST1-FLAG* construct (Figure S2a). Quantitative analysis confirmed strong overexpression in multiple lines (Figure 2a). Phenotypic assessment of two independent lines (*35S::BpHST1::FLAG12* and *35S::BpHST1::FLAG19*) revealed that plant height was significantly reduced compared to the DL-1 (*p* < 0.05), whereas no significant differences were observed in primary branch number and branch angle (Figure 2b–e). To elucidate the cellular mechanism underlying the height reduction, we conducted a histological examination of stem tissues (Figure 2f). The analysis showed that cells in the transgenic lines exhibited a significant decrease in both length and width relative to DL-1 cells (Figure 2g,h). We further investigated the impact of *BpHST1* overexpression on photosynthesis, which revealed that the *35S::BpHST1::FLAG12* line had significantly lower net photosynthetic rate, transpiration rate, and stomatal conductance than DL-1 (Figure 2i, Figure S2b,c). In addition, we found the *35S:BpHST1::FLAG12* line, *BpHST1* also impaired PSII function, marked by decreased Fv/Fm and ΦPSII, and concurrently elevated NPQ for photoprotection. The *35S:BpHST1::FLAG19* line exhibited no significant differences from wild-type DL-1, a result that is likely attributable to the comparatively lower level of transgene overexpression in this line (Figure S2d–f). Collectively, these results demonstrated that *BpHST1* overexpression suppresses plant height and overall growth in birch.

### 3.3. Overexpression of BpHST1 Compromised Light Harvesting Capacity

**Figure 3:**
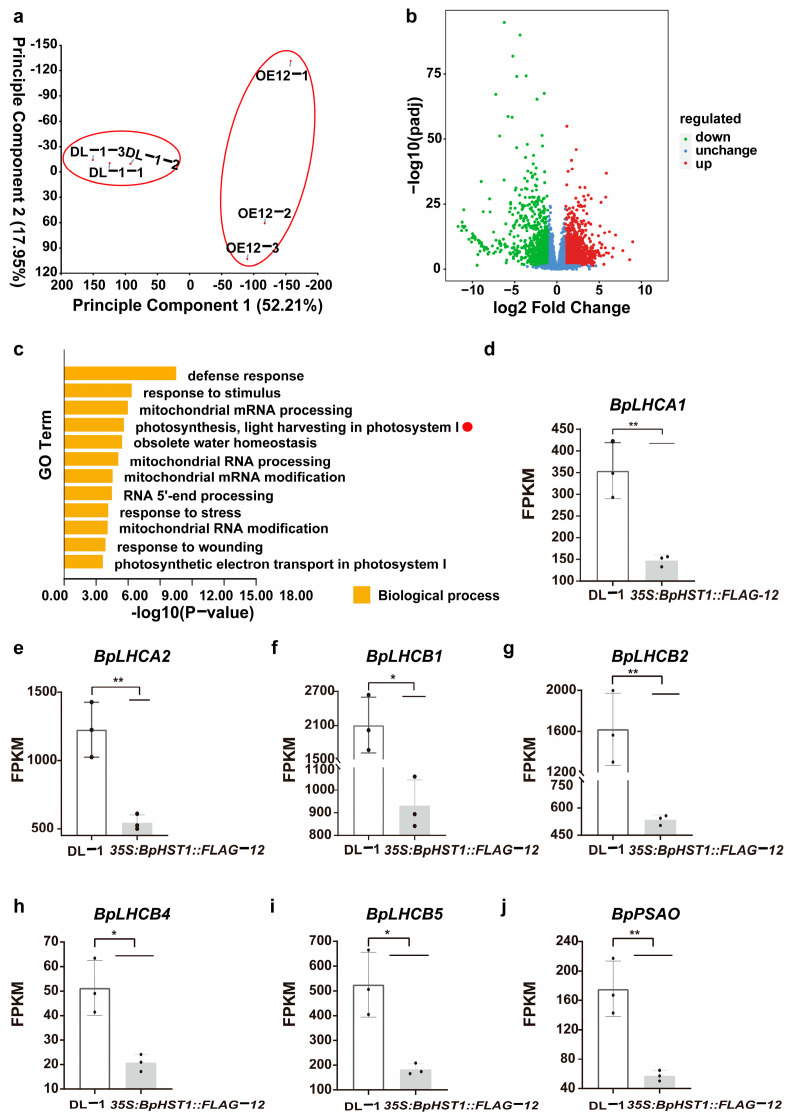
*BpHST1* negatively regulates the expression of key genes in photosynthesis system (**a**) Principal component analysis (PCA) of *BpHST1* transgenic lines and DL-1. (**b**) Volcano plot of *BpHST1* transgenic lines and DL-1. (**c**) Gene Ontology (GO) enrichment analysis of *BpHST1* transgenic lines and DL-1. (**d**–**j**) The FPKM values of all genes in photosynthesis terms in *BpHST1* transgenic lines and DL-1. Data are presented as the mean ± SD, *n* = 3. Asterisks represent significant differences (* *p* < 0.05, ** *p* < 0.01, *t*-test).

To investigate the mechanism of *BpHST1*, we subsequently performed transcriptome sequencing (RNA-seq) from the leaves of 2-month-old DL-1 and the *BpHST1* transgenic line *35S::BpHST1::FLAG12*. Principal component analysis (PCA) of the RNA-seq data showed clear separation between DL-1 and transgenic samples. The data points exhibited distinct intra-group clustering, indicating high reproducibility among biological replicates (Figure 3a). Based on the criteria of expression absolute log2 fold change ≥ 1 and adjusted *p* < 0.01 [43], we identified 1610 significantly upregulated genes and 1364 significantly down regulated genes in *35S::BpHST1::FLAG12* compared to DL-1 (Figure 3b). We identified Gene Ontology (GO) term enrichment analysis of 1364 DEGs was enriched in defense response (*p* < 0.001, GO:0006952), response to stimulus (*p* < 0.001, GO:0050896), mitochondrial mRNA processing (*p* < 0.001, GO:0090615), photosynthesis, light harvesting in photosystem I (*p* < 0.001, GO:0009768), obsolete water homeostasis (*p* < 0.001, GO:0030104), mitochondrial RNA processing (*p* < 0.001, GO:0000963) (Figure 3c). Based on the findings that *BpHST1* is light-responsive and influences photosynthesis (Figure 1c, Figure 2i), we directed our attention to the photosynthesis, light harvesting in photosystem l (GO:0009768). Next, we compared the transcript levels of photosynthetic genes. The results indicated that the expression of photosystem-related genes was significantly downregulated in the transgenic lines compared to DL-1 (Figure 3d–j). Collectively, these findings suggest that *BpHST1* negatively regulates plant height and growth in birch, likely by repressing photosynthetic gene expression and thereby limiting photosynthetic capacity.

### 3.4. BpHST1 Binds to the Promoter of BpLHCA2 and Represses the Expression of Its Downstream Genes

**Figure 4:**
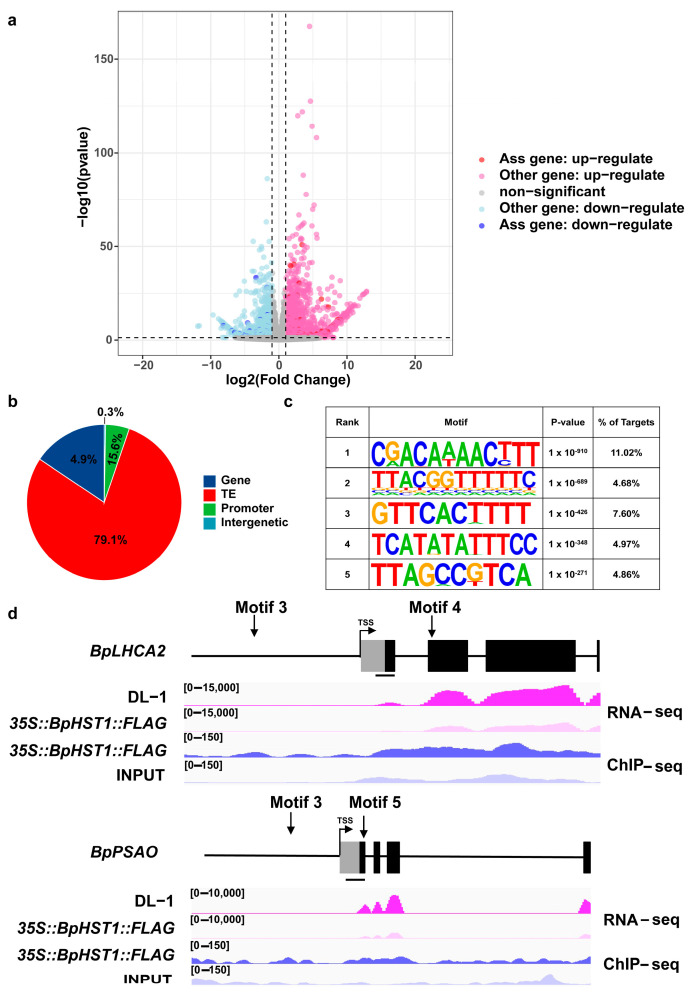
Association analysis of *35S::BpHST1::FLAG* ChIP-seq and RNA-seq. (**a**) Association between *35S::BpHST1::FLAG* genomic binding and transcriptional changes. (**b**) Analysis of *35S::BpHST1::FLAG* binding sites by ChIP-seq. (**c**) Identification of *35S::BpHST1::FLAG* ChIP-seq binding motifs. (**d**) IGV illustrating the reproducible peaks of *35S::BpHST1::FLAG* ChIP-seq and RNA-seq. Grey boxes, black boxes and black lines represent the 5′-UTR, exons and introns, respectively. Transcription start sites were presented with arrows, Bar = 100 bp.

To define the genome-wide binding site of *BpHST1*, we conducted ChIP-seq using a FLAG-tagged *BpHST1* transgenic line and focused on genomic regions with significant binding peaks that overlapped with down-regulated genes (Figure 4a, *p* < 0.01). The DNA binding site of *BpHST1* is dominated by transposable elements, implicating its primary role in epigenetic silencing. Binding events at gene promoters further suggested a capacity for direct transcriptional regulation (Figure 4b). To elucidate the DNA-binding specificity of the BpHST1 protein, we performed motif analysis. The identification of distinct sequence motifs indicated that *BpHST1* recognizes specific DNA patterns to regulate diverse downstream genes, thereby participating in complex biological processes (Figure 4d and Figure S3). IGV showed no significant changes in the repeatable peaks of 35S::BpHST1::FLAG ChIP-seq and RNA-seq of other genes in photosystem I (Figure S3). Binding motifs for *BpHST1* were identified in the promoters of *BpLHCA2* and *BpPSAO*. Accordingly, IGV visualization demonstrated a strong correlation between *BpHST1::FLAG* enrichment (ChIP-seq) and transcriptional repression (RNA-seq) at these loci, supporting their direct regulation by *BpHST1*. Protein-DNA docking predictions via AlphaFold Server 3 suggested that *BpHST1* binds the *BpLHCA2* promoter through specific hydrogen bonds, indicating potential for direct physical interaction. This result supports the theory in which BpHST1 exerts its function through possible binding to the *BpLHCA2* promoter, thereby mediating transcriptional repression (Figure S4). Based on these findings, we proposed that *BpLHCA2* functions as a direct downstream target of *BpHST1*, mediating its regulation of plant growth and development.

## 4. Discussion

### 4.1. BpHST1 Acted as a Downstream Effector of Light Signaling in Birch

*BpHST1* expression was light-responsive, a property potentially linked to light-regulatory cis-elements in its promoter (Figure S1a), with transcript levels decreasing in darkness and increasing under light (Figure 1c). Interestingly, this result is consistent with the expression pattern of the *HST1* gene in *Arabidopsis thaliana*, which is highly expressed under light conditions [44]. This might be due to both birch and *Arabidopsis* being dicotyledon plants. In contrast, in rice, as a monocot plant, the *HST1* gene exhibits low expression under light and high expression in darkness [45]. Despite these divergent expression patterns, *HST1* homologs both mediate light signal responses, reflecting functional conservation of the gene. The functional and evolutionary divergence of *HST1* between monocot and dicot plants requires further investigation.

### 4.2. BpHST1 Mediated Repression of Light-Harvesting Gene BpLHCA2

Integrated RNA-seq and ChIP-seq analyses demonstrated that *BpHST1* overexpression downregulates the PSI light-harvesting pathway and directly targets *BpLHCA2*. As one of the core subunits of the light-harvesting chlorophyll a/b complex I (LHCI) associated with photosystem I (PSI), *LHCA2* is widely present in photosynthetic organisms such as green algae, mosses, and higher plants [46]. Its core functions encompass participating in light energy absorption, transfer, and the regulation of photosynthetic electron flow, making it a critical component that links the light reaction to energy conversion in the photosynthetic system [47]. Specifically in higher plants, *LHCA2* is involved in the assembly of the PSI-LHCII complex, where the phosphorylation site at its N-terminus serves as a key determinant for LHCII binding during state transitions [48]. Furthermore, by binding to chlorophylls, *LHCA2* expands the light absorption range of the photosynthetic system, efficiently transfers the captured light energy to the PSI reaction center, and optimizes energy utilization under low-light conditions [49]. Collectively, these characteristics highlight that *LHCA2* plays crucial functional and applicative roles in the regulation of photosynthetic efficiency in higher plants [34,50]. The transgenic lines also exhibited reduced photosynthetic rates and stomatal conductance, indicating additional effects on electron transport (Figure 3c and Figure 4c). The importance of this pathway for plant architecture is further supported by findings in rice, where mutation of *OsPS1-F* (*Oryza sativa PHOTOSYSTEM 1-F*) caused significant developmental defects, including altered plant height and tiller number [51]. Collectively, this study provided new evidence for an “epigenetic–photosynthesis–growth” regulatory network and highlight a potential target for epigenetic breeding in forest trees.

### 4.3. The Recruitment Mechanism of BpHST1

Our study reveals that *BpHST1*, a class of histone deacetylase, likely influences plant architecture and represses downstream gene expression by modulating histone modifications (Figure 1a, Figure 2b and Figure 4). However, the precise mechanism by which *HST1* is recruited to its genomic targets remains unclear. Transcriptional regulators are known to play a key role in directing histone deacetylases to specific genomic loci [52]. For instance, transcription factors such as MYB-like proteins can recruit corepressors like *TOPLESS* along with the histone deacetylase *HDA19* to form a repressive complex that downregulates *miR159* expression by reducing H3K9ac levels at its promoter [53]. HDA19-mediated histone deacetylation suppresses the transactivation capacity of the defense-related transcription factors *WRKY38/62*, establishing an epigenetic layer in immune response regulation [54]. Beyond transcription factors, other chromatin-associated proteins—including chromatin remodelers such as SWI/SNF and epigenetic modifiers like methyltransferases—may also contribute to the recruitment of *HST1* [55,56]. Therefore, whether additional transcription factors or interacting proteins participate in the molecular mechanism by which *HST1* regulates plant architecture warrants further investigation.

## 5. Conclusions

In summary, our study established *BpHST1* as a key epigenetic regulator governing plant architecture in birch through the modulation of photosynthetic pathways. We demonstrated that *BpHST1*, which shows light-responsive and leaf-predominant expression, significantly affects the plant architecture when overexpressed in birch, leading to reduced plant height and cell size, and impaired photosynthetic capacity. Combined with RNA-seq and ChIP-seq association analysis, we postulated that *BpLHCA2* may function as a downstream targeted gene of *BpHST1*, mediating plant growth and development. These findings offer a novel insight into the epigenetic control of forest architecture and provide a valuable foundation for future epigenetic breeding strategies aimed at improving forest productivity and adaptation.

## Data Availability

The raw data supporting the conclusions of this article will be made available by the authors on request.

## Supplementary Materials

The following supporting information can be downloaded at: https://www.mdpi.com/article/10.3390/biology14121689/s1, Figure S1: Analysis of *BpHST1* promoter and BpHST1 protein features, and the vector construction of the *BpHST1* promoter.; Figure S2: Construction of the *BpHST1* overexpression vector and the photosynthetic physiological analysis; Figure S3: IGV illustrating the reproducible peaks of *35S::BpHST1::FLAG* ChIP-seq and RNA-seq; Figure S4: AlphaFold3-predicted binding of BpHST1 to *BpLHCA2* promoter regions. Table S1: Primer design of PCR.
