# Supplementary material for: Histone Deacetylase BpHST1 Regulates Plant Architecture and Photosynthesis in Birch
Source: Biology (Basel). 2025 Nov 27;14(12):1689. doi: 10.3390/biology14121689 (PMC12729910; doi:10.3390/biology14121689)
Supplement: Supplementary file 1 [file biology-14-01689-s001.zip › biology-3991998-supplementary.pdf]

Supplementary Figure

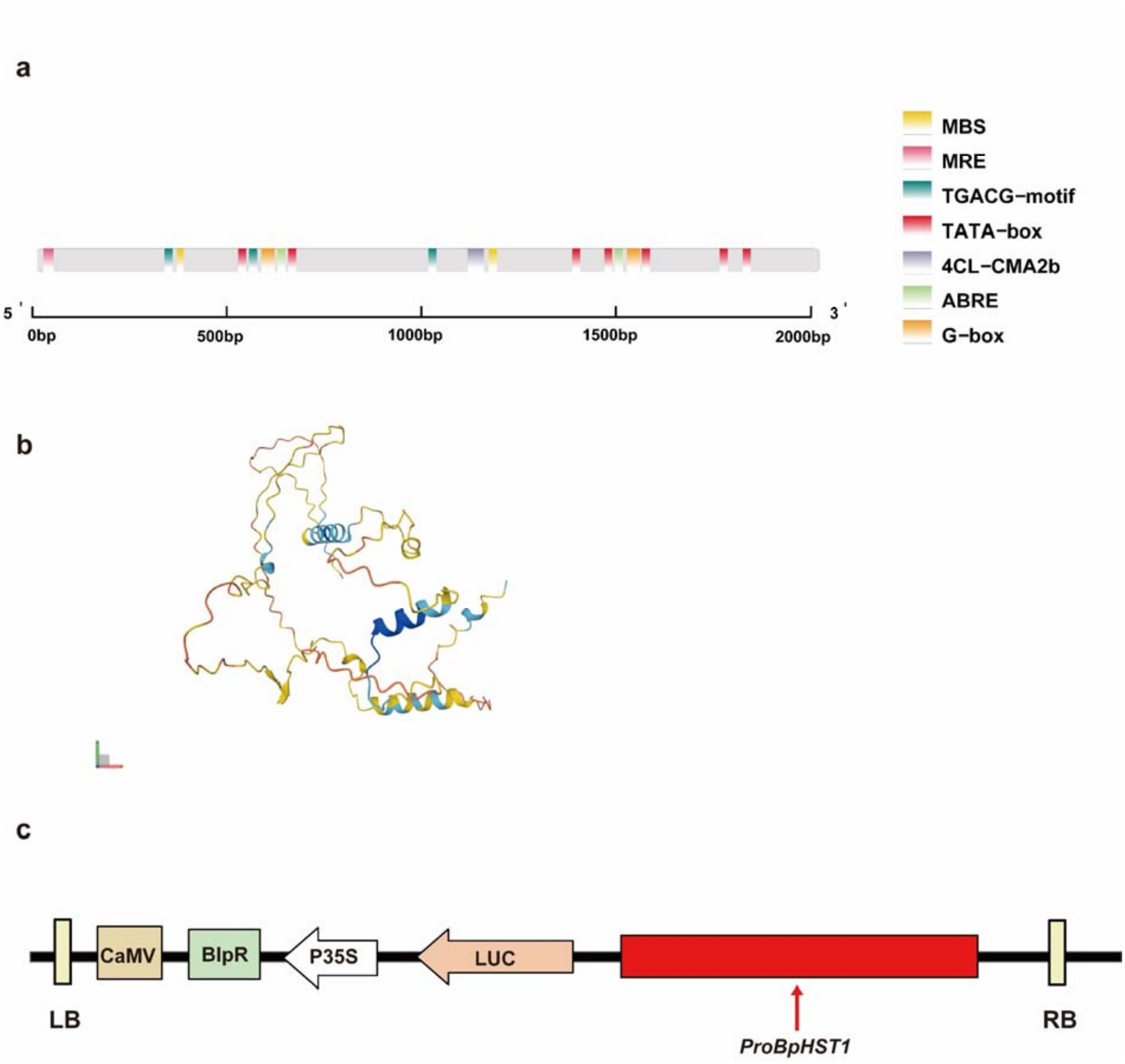

Figure S1. Analysis of *BpHST1* promoter and BpHST1 protein features, and the vector construction of the *BpHST1* promoter. (a) Analysis of cis–acting elements in the *BpHST1* promoter. (b) Predicted structure characteristics of the BpHST1 protein. (c) Schematic representation of the *BpHST1* promoter vector.

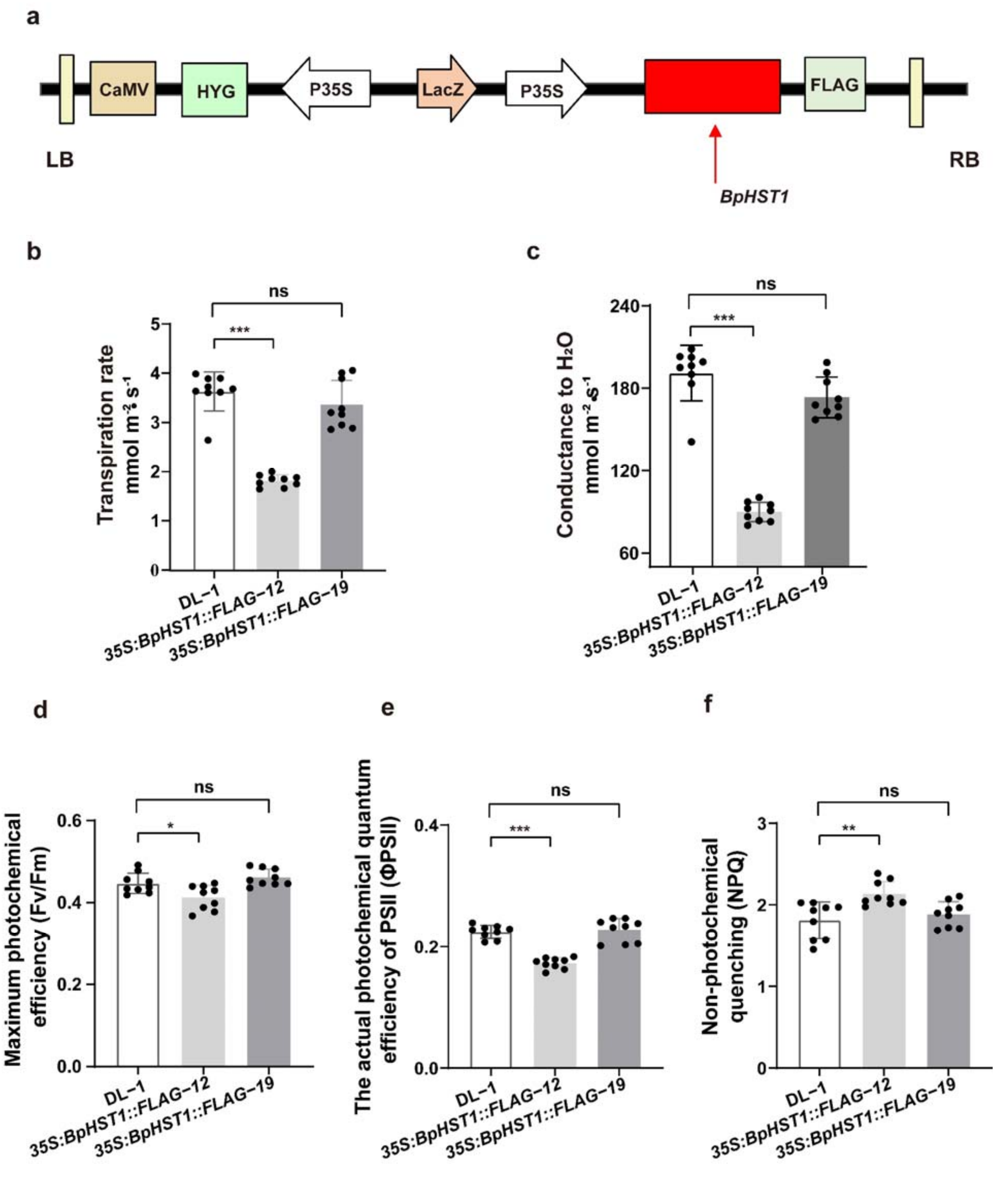

Figure S2. Construction of the *BpHST1* overexpression vector and the photosynthetic physiological analysis. (a) Schematic diagram of the structure of *BpHST1* overexpression vector. (b) Transpiration rate. (c) Conductance to H_2O. (d, e, f) Maximum photochemical efficiency of PSII (Fv/Fm), Actual photochemical efficiency of PSII (ΦPSII) and Non-photochemical quenching (NPQ).

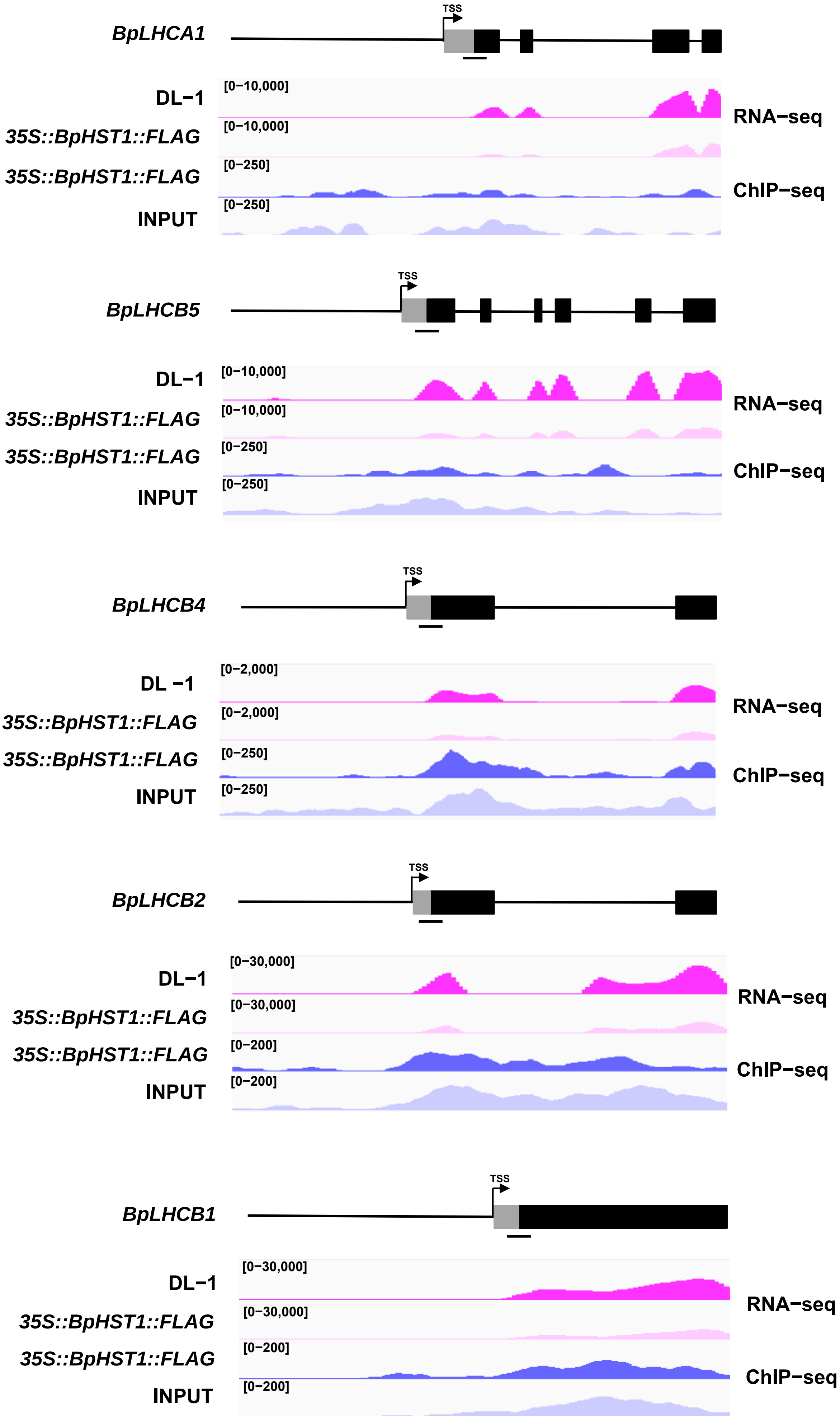

Figure S3. IGV illustrating the reproducible peaks of *35S::BpHST1::FLAG* ChIP−seq and RNA−seq. Grey boxes, black boxes and black lines represent the 5′−UTR, exons and introns, respectively. Transcription start sites were presented with arrows, Bar=100 bp.

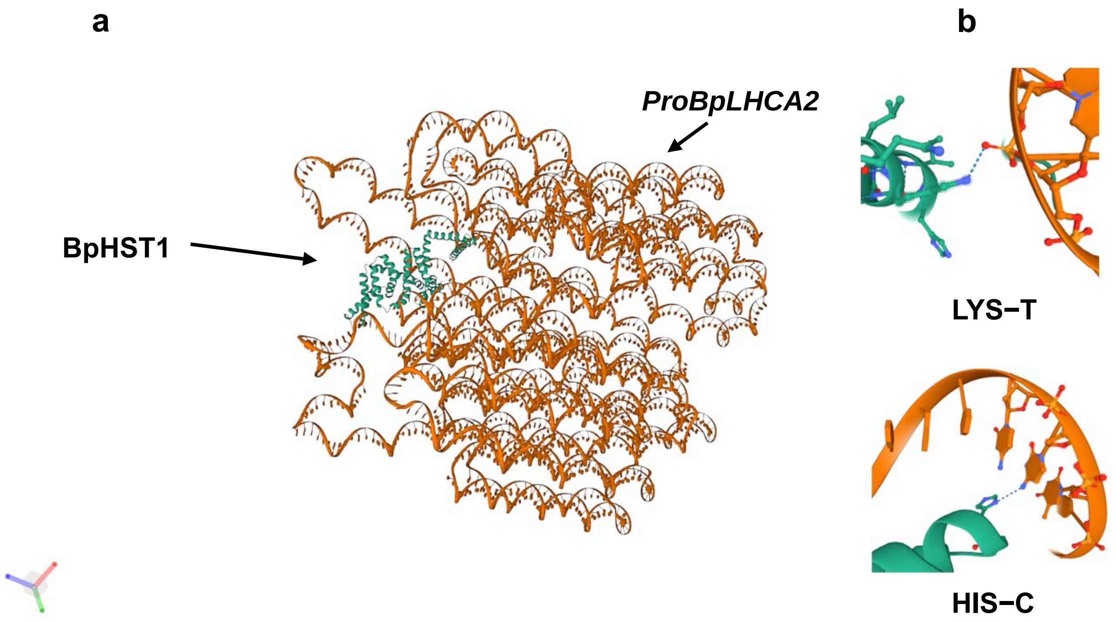

Figure S4. AlphaFold3–predicted binding of BpHST1 to *BpLHCA2* promoter regions. (a) Structural overview of BpHST1 and *BpLHCA2* promoter. (b) Hydrogen bonding network between BpHST1 and *BpLHCA2* promoter. LYS: Lysine; HIS: Histidine.

Supplemental Table S1. Primer design of PCR.

Supplemental Table S1 Primer design of PCR.

Primer	Primer sequence
TUB–F	AACTCATCAGCGGCAAGGAA
TUB–R	GTAGAAAGGACGCTGTTGTAGGG
HST1–qRTF	ACCGTCCTTAAGGATGGGTTAGC
HST1–qRTR	TCGTTGTCATCATCGCCACCG
HST1–QF	ATGAAGATCTTCAACTGGGTACATAGG
HST1–QR	GACTGTAGCACCTTGAGTTGGAA
ProHST1–F	GATCAATTGGTGTGCATTGAGC
ProHST1–R	AATCAGGTGAAGCCACGTGTCC
1301F	GCGCAACTGCAACTCCGATAA
